# Genome-Wide Identification and Characterization of bZIP Transcription Factors in *Brassica oleracea* under Cold Stress

**DOI:** 10.1155/2016/4376598

**Published:** 2016-05-23

**Authors:** Indeok Hwang, Ranjith Kumar Manoharan, Jong-Goo Kang, Mi-Young Chung, Young-Wook Kim, Ill-Sup Nou

**Affiliations:** ^1^Department of Horticulture, Sunchon National University, 255 Jungang-ro, Suncheon, Jeonam 57922, Republic of Korea; ^2^Department of Agricultural Education, Sunchon National University, 255 Jungang-ro, Suncheon, Jeonam 57922, Republic of Korea

## Abstract

Cabbages (*Brassica oleracea* L.) are an important vegetable crop around world, and cold temperature is among the most significant abiotic stresses causing agricultural losses, especially in cabbage crops. Plant bZIP transcription factors play diverse roles in biotic/abiotic stress responses. In this study, 119 putative BolbZIP transcription factors were identified using amino acid sequences from several bZIP domain consensus sequences. The BolbZIP members were classified into 63 categories based on amino acid sequence similarity and were also compared with BrbZIP and AtbZIP transcription factors. Based on this BolbZIP identification and classification, cold stress-responsive* BolbZIP* genes were screened in inbred lines,* BN106* and* BN107*, using RNA sequencing data and qRT-PCR. The expression level of the 3 genes,* Bol008071*,* Bol033132*, and* Bol042729*, was significantly increased in* BN107* under cold conditions and was unchanged in* BN106*. The upregulation of these genes in* BN107*, a cold-susceptible inbred line, suggests that they might be significant components in the cold response. Among three identified genes,* Bol033132* has 97% sequence similarity to* Bra020735*, which was identified in a screen for cold-related genes in* B. rapa* and a protein containing N-rich regions in LCRs. The results obtained in this study provide valuable information for understanding the potential function of BolbZIP transcription factors in cold stress responses.

## 1. Introduction

Cabbage (*Brassica oleracea* L.) plants represent one of the major vegetable crops grown worldwide. Most crops of* B. oleracea* and its sister species* Brassica rapa* produce a range of phytochemicals with diverse functions for plant defense such as polyphenolic compounds, carotenoids, and glucosinolates [1, 2]. The draft genome sequences of* B. oleracea* (with the CC genome) and* B. rapa* (with the AA genome) were recently published [3, 4]. A total of 66.5% (34,237) of* B. oleracea* genes and 74.9% (34,324) of* B. rapa* genes were clustered. In total, 5,735* B. rapa-*specific genes and 9,832* B. oleracea*-specific genes among 45,758 protein coding genes were identified. The availability of published genome sequence for these crop plants facilitates studies of structural and functional genomics in agronomically important species.

Plant bZIP transcription factors play diverse roles in developmental and physiological processes and biotic/abiotic stress responses such as ABA signaling for osmotic stress responses during vegetative growth [5], seed germination and flowering time [6], glucose-ABA signaling [7], sugar signaling during metabolism [8], lipid stress responses [9], response to zinc deficiency [10], salicylic acid- (SA-) dependent plant systemic defense responses and the activation of jasmonic acid- (JA-) and ethylene (ET-) dependent defense mechanisms [11], anthocyanin accumulation during photo morphogenesis [12], floral patterning [13], auxin-mediated histone acetylation related AtbZIP11 [14], and ABA signaling related to stress tolerance [15]. As the focus of recent studies due to their importance as regulator of responses to the biotic and abiotic stresses, bZIP transcription factors have been identified in diverse plants. Based on the presence of the UARR and LCRs, 136 bZIPs were identified in* B. rapa*; 64 were found in cucumber based on predicted structural features, 92 in sorghum through genome-wide identification and characterization, 89 in rice according to their DNA binding specificity and amino acid sequences in basic and hinge regions, 131 in soybean based on the basic region of the bZIP domain and the presence of additional conserved motifs, 75 in* Arabidopsis* according to sequence similarities of their basic region and additional conserved motifs, and 141 in* Hordeum vulgare* [16–22]. However, little is known about the genome-wide survey and expression patterns of bZIP transcription factors in* B. oleracea*. Among the BolbZIPs, the function of only one gene related with drought stress and ABA has been reported. Expression of* BolABI5* was dramatically induced by drought stress and exogenous ABA [23]. Heterogeneous expression of* BolABI5* rescued the ABA-insensitive phenotype of the* Arabidopsis abi5-1* mutant during seed germination, suggesting that* BolABI5* likely functions in positive regulation of plant ABA responses.

The bZIP domain includes a basic region and a leucine zipper located on a contiguous *α*-helix. An N-x7-R/K motif comprising ~16 amino acids constitutes the basic region, which binds DNA containing a nuclear localization signal. The leucine zipper is composed of leucine residue repeat and is positioned precisely at nine amino acids towards the C-terminus from the arginine in the basic region, creating an amphipathic helix. To bind DNA, two subunits adhere via interactions between the hydrophobic sides of their helices, which create a superimposed coiled-coil structure for homo- or/and heterodimerization. Plant bZIPs preferentially bind to specific sequences, namely, the A-box (TACGTA), C-box (GACGTC), and G-box (CACGTG), but there are also examples of nonpalindromic binding sites [21].

In this study, we identified 119 BolbZIP proteins using the consensus sequence of several bZIP proteins and classified them based on specific amino acid sequence, unique amino acid repeat regions (UARRs), and low complexity regions (LCRs). Additionally, transcriptome analysis related to cold stress responses using RNA sequencing provided valuable information for research into stress tolerance and molecular breeding in* B. oleracea*.

## 2. Materials and Methods

### 2.1. Database Searches for bZIP Transcription Factors in* B. oleracea*

The AtbZIP, BrbZIP, and BolbZIP amino acid sequences obtained from TAIR (http://www.arabidopsis.org/), BRAD (http://brassicadb.org/brad/), and Bolbase (http://ocri-genomics.org/bolbase/). To confirm the presence of bZIP domain, UARR and LCRs in putative AtbZIP, and BrbZIP and BolbZIP proteins, the Motif scan tool (http://myhits.isb-sib.ch/cgi-bin/motif_scan), SMART tool (http://smart.embl-heidelberg.de/), and Batch CD-search tool (http://www.ncbi.nlm.nih.gov/Structure/bwrpsb/bwrpsb.cgi) were used. bZIP proteins that showed the presence of a bZIP domain, UARR, and LCRs with confidence (*E*-value < 0.1) in the Motif scan tool and Batch CD-search tool were used for further analyses. Next, LCRs were identified using the SMART tool.

### 2.2. Plant Material and Cold Treatment

Seeds of* B. oleracea* (inbred lines “*BN106*” and “*BN107*”) were germinated in soil and then grown for approximately 3 weeks in a growth chamber at 25°C under long day condition (16 h day/8 h night). For cold treatment, the 5-week-old plants were transferred to a 4°C growth chamber under continuous light conditions. The plants were then treated with cold temperature at 4°C for 6 h, followed by 0°C for 2 h. Further, the plants were subjected to freezing treatment at −2°C for 2 h followed by 4°C for 6 h.

### 2.3. RNA Extraction and cDNA Synthesis

Total RNA was isolated from plant tissues using an RNA extraction kit (Qiagen, USA) according to the manufacturer's protocol. Total RNA was treated with RNase-free DNase (Promega, USA) to remove the genomic DNA contamination. The quality of total RNA was checked using a nanoDrop Spectrometer (nD-1000 Spectrophotometer, Peqlab) and agarose gel electrophoresis. cDNA was then synthesized using Superscript II reverse-transcriptase (Invitrogen), after which 5 *μ*L (about 2 *μ*g) total RNA and 1 *μ*L of oligo dT (500 *μ*g/mL) were mixed in the reaction tube and then heated at 65°C for 10 min. The enzyme was then added into the tube and incubated at 42°C for 50 min. Finally, the reaction tube was incubated at 70°C for 15 min to inactivate the enzyme.

### 2.4. RNA Sequencing

Two cabbage lines,* BN106* and* BN107* which exhibit different sensitivity to cold stress, were used for RNA sequencing. Total RNA was extracted from leaves of* BN106* and* BN107* at 2 h in 0°C. The total RNA was isolated using TRIzol reagent (Invitrogen, USA) following the manufacturer's instructions. Total RNA (20 *μ*g) from each sample, BN106_22°C and BN107_22°C (control) and BN106_0°C and BN107_0°C (treated), were used for Illumina sequencing (33 G 101 bp paired-end reads; Seeders, Republic of Korea). Transcripts of unigenes assembled from the total reads were validated by direct comparison with gene sequences in the Phytozome 15 (https://phytozome.jgi.doe.gov/pz/portal.html) using BLASTx (threshold *E*-value ≤ 1*e* ^−10^). The number of mapped clean reads for each unigene was counted and normalized using the DESeq package in R on two independent biological replicates. From the differentially expressed gene dataset, the transcripts of bZIP transcription factors were analyzed for up- and downregulated differentially expressed genes. BolbZIP sequence and RNAseq database sequences were aligned to each other using ClustalW with default parameters (http://www.genome.jp/tools/clustalw/).

### 2.5. RT-PCR and qRT-PCR

Quantitative real-time PCR (qRT-PCR) and reverse transcription PCR (RT-PCR) were conducted using cDNA from cold treated plants using primers specific for the* BolbZIP* gene (see Table S1 in Supplementary Material available online at http://dx.doi.org/10.1155/2016/4376598). RT-PCR was conducted using cDNA of plants exposed to cold and freezing temperatures (22°C, 4°C, 0°C, and −2°C). The PCR procedure involved predenaturation at 95°C for 5 min followed by cycles of denaturation at 95°C for 30 s, annealing at 60°C for 30 s, extension at 72°C for 30 min, and then a final extension for 5 min at 72°C. qRT-PCR was conducted by subjecting the samples to initial denaturation at 95°C for 10 min followed by 40 cycles of 95°C for 20 s, 60°C for 20 s, 72°C for 30 s, and final extension at 72°C for 2 min. An* actin* primer set for* B. oleracea* was used for normalization of RT-PCR and qRT-PCR.

## 3. Results

### 3.1. Identification of bZIP Transcription Factors in* B. oleracea*

To search for bZIP transcription factors in* B. oleracea*, we used the conserved bZIP domain consensus sequences (Table S2) of several proteins as BLASTP queries against the* Brassica* database (http://brassicadb.org/brad/). In addition, homology searches using 136 BrbZIP proteins were performed [16]. A total of 126 BolbZIP candidates were initially obtained with a probability *E*-value threshold of 0.05. To confirm the presence of a bZIP domain in the selected bZIP proteins, domain searches were performed with several tools (see Section 2). After exclusion of the proteins lacking a bZIP domain, 119 putative BolbZIP transcription factors were identified. The position of each candidate* BolbZIP* gene in* B. oleracea* chromosome data available at Bolbase (Version 1.0) was then determined.

**Figure 1:**
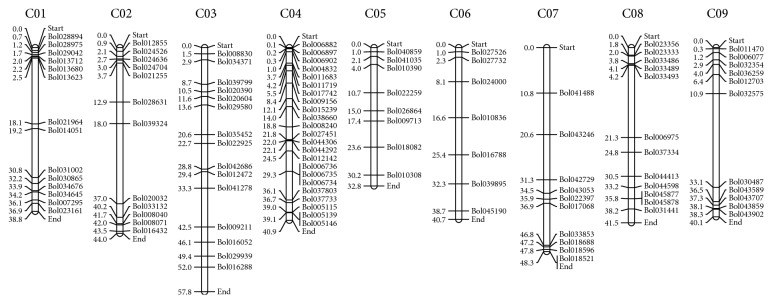
Distribution of* BolbZIP* genes onto the nine assembled* B. oleracea* chromosomes. Graphical (scaled) representation of physical locations for each* BolbZIP* gene on* B. oleracea* chromosomes (numbered C01–C09). Chromosomal distances are given in Mbp.

Among 119 candidate* BolbZIP* genes, 112 were mapped on chromosomes C01–C09 (Figure 1). 14 genes of* BolbZIP* were mapped on C01, 12 genes on C02, 15 genes on C03, 23 genes on C04, 8 genes on C05, 7 genes on C06, 10 genes on C07, 12 genes on C08, and 11 genes on C09. In particular, 20% of the* BolbZIP* genes mapped to chromosome 4 (Table S3). In addition, 7 genes were found in scaffolds that have yet been mapped to chromosomes.* Bol024237* was anchored on Scaffold000093,* Bol019052* on Scaffold000133,* Bol016607* on Scaffold000153,* Bol004200* on Scaffold000329,* Bol003614* on Scaffold000345,* Bol001886* on Scaffold000417, and* Bol000879* on Scaffold000492.

### 3.2. Classification of BolbZIP Transcription Factors

We have classified the BolbZIP transcription factors based on amino acid sequence similarity to 136 BrbZIP and 75 AtbZIP proteins previously reported (Table 1) [16]. For the majority of bZIP proteins, we found orthologous groups including counterparts from each species, although occasionally no BrbZIP or AtbZIP homologs were found. AtbZIP and BrbZIP homologs of the BolbZIP proteins are summarized in Table 1. The proteins were divided into 63 categories based on the amino acid sequence similarity (Table 1). Most categories included two or three BolbZIP and BrbZIP proteins but a single AtbZIP. Analysis of the amino acid sequences revealed that the similarity between BolbZIP, BrbZIP, and AtbZIPs ranged from 50% to 90%. Several BolbZIP proteins showed over 90% similarity to the corresponding AtbZIP. For example, the similarity among Bol010308, At3g12250, and At5g06950 was 91–94%. For other genes, the closest homologs (with over 90% amino acid homology) were between the BolbZIP and the BrbZIP such as Bol004832 and Bra004689. BolbZIP proteins were also classified according to the method by Hwang et al. [16] based on UARRs and LCRs, which were further divided into 9 groups: glutamine (Q), aspartic acid (D), proline (P), asparagine (N), serine (S), glycine (G) rich domain, transmembrane (Tm) domain, LCRs only, and no LCRs except bZIP domain (Table 2, Tables S4 and S5). BolbZIP proteins and their orthologs from* B. rapa* and* A. thaliana* were found in the same groups. For example, BolbZIP of category 1 and its homologs Bra004550 and At2g46270 were classified into group 3A. LCRs of group 11 (only LCRs present) bZIP proteins composed single and mixed repeat natural amino acids. Group 12 contained bZIP proteins with no LCRs or specific amino acid-rich regions.

### 3.3. Candidate* BolbZIP* Genes for Responses to Cold Stress

**Figure 2:**
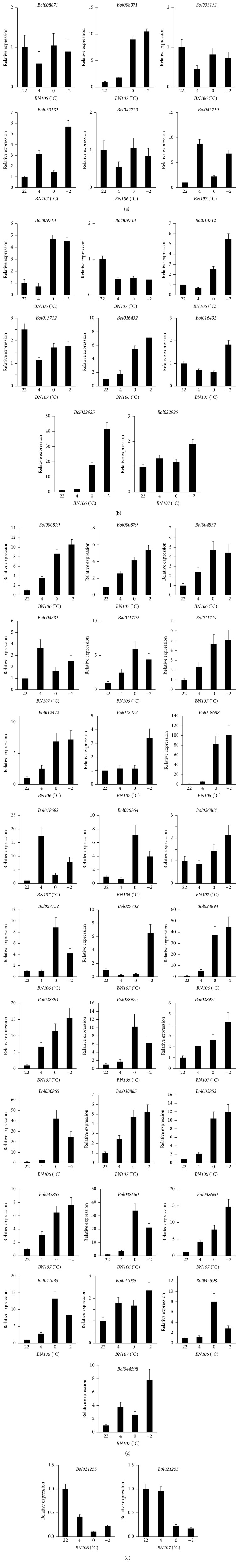
Relative expression levels of 22* BolbZIP* genes in cabbage inbred lines cold-tolerant* BN106* and cold-susceptible* BN107* under cold stress conditions. 5-week-old plants were treated at 4°C, 0°C, and −2°C. The* actin* transcript levels were used for normalization. Values shown are relative to transcript levels in the 22°C treated plants. Error bars indicate standard deviation. (a) Genes showing no significant relative expression change in* BN106* and upregulating at all temperatures in* BN107*. (b) Genes showing upregulation at all temperatures in* BN106* and no significant relative expression change in* BN107*. (c) Genes showing greater upregulation at lower temperatures in* BN106* and* BN107*. (d) Genes showing downregulation in response to cold in* BN106* and* BN107*.

**Figure 3:**
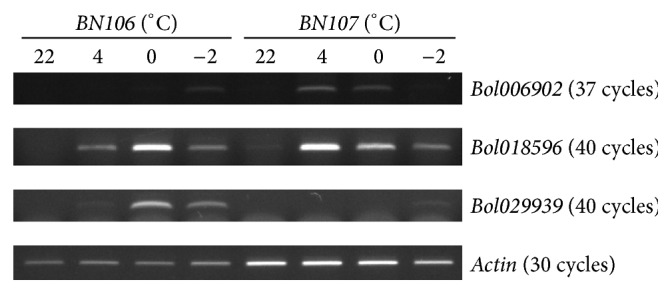
RT-PCR analysis of three* BolbZIP* genes in response to cold. These genes showed no expression in 22°C-treated cabbage inbred lines* BN106* and* BN107*. The* actin* transcript levels were used as an internal control.

**Figure 4:**
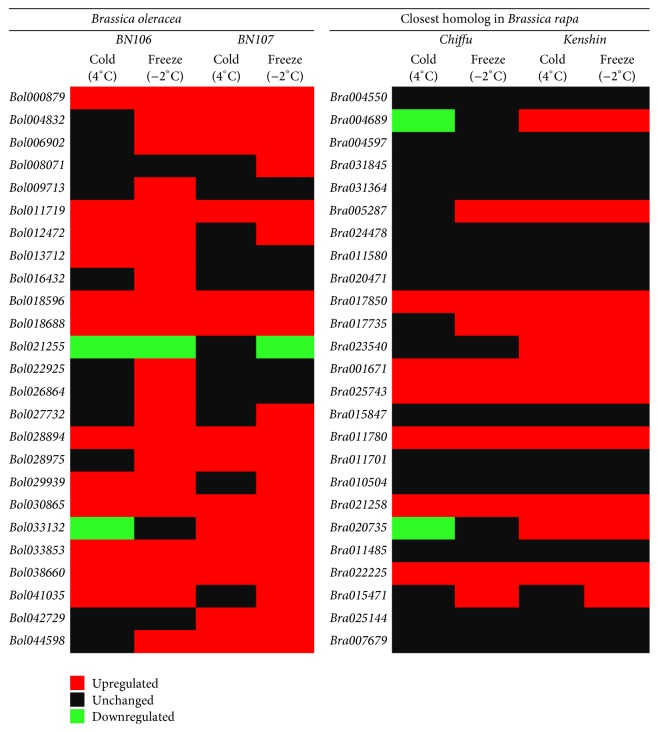
Heat map representation of cold-responsive expression of* BolbZIP* and* BrbZIP* genes. The expression pattern of the BolbZIPs and their closest BrbZIP homologs in response to cold (4°C) and freezing (−2°C) stresses are shown. Heat map was generated using up- and downregulated gene expression data from qRT-PCR and RT-PCR results.

**Figure 5:**
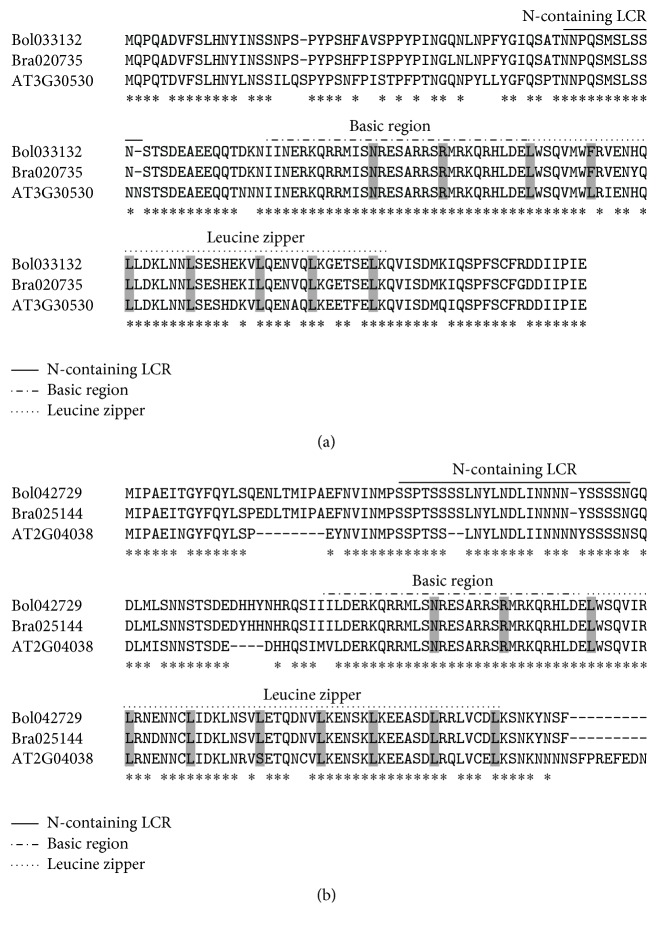
Amino acid sequences of Bol003312 and Bol042749 and their homologs. (a) An alignment of the amino acid sequences of Bol033132 and two homologs, Bra020735 and At3g30530. Conserved sequences of bZIP domain are highlighted using gray shade in the basic and leucine zipper regions. (b) An alignment of the amino acid sequences of Bol042729 and two homologs, Bra025144 and At2g04038.

To identify BolbZIP genes that might function in responses to cold stress, we carried out comparative analysis of the expression of* BolbZIP* gene in two* B. oleracea* inbred lines, cold-tolerant* BN106* and cold-susceptible* BN107*.* BolbZIP* genes were selected from an RNA sequencing dataset based on their annotations and their expression profiles were analyzed (data not shown). Among the 119* BolbZIP* genes, the expression of 41 genes was remarkably changed in responses to cold treatment, whereas 78 genes of them showed no significant changes in their expression.* BolbZIP* genes with significantly different expression were determined in 4°C-treated sample base on fold change (FC) ≥2 and ≤0.5 relative to 22°C-treated sample. Cold treatment at this temperature caused the upregulation of 18 genes in* BN106* and of 7 genes in* BN107*, whereas 15 genes were downregulated in* BN106* and 8 genes were in* BN107* by cold treatment. In total, the expression of 21 genes was upregulated and 20 genes downregulated by cold treatment (Table 3). In addition, 6 genes were not showing any expression within* BN106* lines and therefore not calculated (Table 3). Finally, 47* BolbZIP* genes' expression level was confirmed using quantitative real-time PCR (qRT-PCR) (Table 3). To obtain detailed expression for the putative cold-response BolbZIP genes thus identified, qRT-PCR was carried out using samples from plants treated at several temperatures (22°C, 4°C, 0°C, or −2°C). Totally, 25* BolbZIP* genes with significantly different expression were selected based on fold-changes (FC) ≥3 and ≤0.5 relative to the control sample (22°C). Most of the tested genes were significantly upregulated by cold treatment except* Bol021255*. Among 25 tested genes, 22 genes are displayed in Figure 2 and three genes by RT-PCR in Figure 3. We were not able to determine the analogous relative expression for the latter three genes because they were not expressed in the 22°C treated sample. The expression levels of several* BolbZIP* genes were comparable between the two lines. However, no significant change in the expression of* Bol008071, Bol033132, and Bol042729* was observed in response to cold treatment in* BN106*, whereas these genes were upregulated at all temperatures in* BN107* (Figure 2(a)). By contrast,* Bol009713, Bol013712, Bol016432, and Bol022925* were upregulated in* BN106*, but not in* BN107* (Figure 2(b)). The increased expression of* 17 BolbZIP* genes was more pronounced after severe cold treatment at 4°C, 0°C, and −2°C (Figure 2(c)) and one gene was downregulated by cold treatment in both* BN106* and* BN107* (Figure 2(d)). Homologs of cold stress-response* BrbZIP* genes were included in the qRT-PCR [16]. These expression patterns are summarized in Figure 4. Moreover, several genes including* Bol016432*,* Bol022925*,* Bol026864*,* Bol027732*, and* Bol028975* displayed differential expression between cold (4°C) and freezing (−2°C) temperature. The expression level of the 3 genes,* Bol008071*,* Bol033132*, and* Bol042729*, was significantly increased in* BN107* under cold conditions and was unchanged in* BN106*. Among three genes,* Bol033132* has 97% sequence similarity to* Bra020735* which was previously reported gene. Two proteins, Bol033132 and Bra020735, contained N-rich regions in LCRs (Figure 5(a)). Moreover, Bol042729 included the N-containing LCR (Figure 5(b)). We suggest the possibility that BolbZIP proteins as well as BrbZIP proteins containing N-rich regions might be involved in cold stress response.

## 4. Discussion

It was known that* B. rapa* and* B. oleracea* genomes are highly similar in their gene structure, but there still exist species-specific genes in two species. Hence this study was carried out in* B. oleracea* and identified 119 BolbZIP proteins and placed them into 63 categories according to sequence similarity (Table 1). To identify the bZIP proteins in* B. oleracea*, a few bZIP domain consensus sequences of several species were used (Table S2). It is possible that this approach could lead us to underestimate the number of bZIP proteins present, despite the high number of BolbZIP proteins we identified. To address this, other search methods or more detailed consensus sequences for bZIP proteins in plants could be examined. In* Arabidopsis*, bZIP proteins were classified into different groups and subfamilies according to sequence similarities in their basic region and additional conserved motifs in order to elucidate the likely function of the proteins [21]. In rice, Nijhawan et al. [19] published 89 bZIP transcription factor-encoding genes based on DNA binding specificity and amino acid sequences in basic and hinge regions. Recently BrbZIP and AtbZIP proteins were divided into 9 groups based on their UARR and LCRs, which are highly enriched in one or a few amino acids [16]. In this study, 119 BolbZIP proteins were categorized into 63 groups and also classified according to UARR and LCRs based on the classification method of Hwang et al. [16]. In addition, the sequence similarity of the bZIP proteins of* B. oleracea*,* B. rapa,* and* A. thaliana* was analyzed. Most of homologs were found to have the same UARR and LCRs. UARRs were composed of 6 amino acids including Q, D, P, N, S, and G in the* B. oleracea* (Tables 2 and S4). This conservation of amino acid composition suggests that these 6 amino acids are important for biological functions and formation of protein structures in bZIP proteins.

*BolbZIP* gene family members were physically mapped to all the nine chromosomes of* B. oleracea*. Among them, chromosome 04 was found to contain the highest number of* BolbZIP* genes (21%), while chromosomes 05 and 06 harbored the fewest (6-7%) (Figure 1, Table S3). In* B. rapa*, the highest number of* BrbZIP* genes was detected in chromosome 09 (21%) [16]. Additionally, most* BolbZIP* genes were distributed in the arm end of each chromosome. The clustered distribution pattern of the* BolbZIP* genes on some chromosomes might be indicated in significant regions evolutionarily. For example,* BolbZIP* genes located on chromosomes 01, 02, 04, 07, and 08, and chromosomes 09 appear to be clustered at the arm end in those chromosomes (Figure 1).

To screen for cold stress-responsive* BolbZIP* genes, we tested the transcription patterns of* BolbZIP* genes enhanced or decreased by cold treatment in two* B. oleracea* lines that showed different cold tolerance [16]. Based on their expression patterns, the cold-responsive* BolbZIP* transcription factors were divided into four groups (Figure 2). We found that the expression of three genes,* Bol008071*,* Bol033132,* and* Bol042729*, was upregulated in cold-susceptible* BN107* but not changed in cold-tolerant* BN106*. Additionally, when compared with 6 genes published for significant BrbZIP factors involved in the cold response, 4* BolbZIP* genes (*Bol004832*, homologous to* Bra000256, Bra004689,* and* Bra003320*;* Bol033132*, homologous to* Bra020735*;* Bol018688*, homologous to* Bra011648*; and* Bol021255*, homologous to* Bra023540*) showed similar patterns of expression in response to cold treatment. For example,* Bol033132* showed an expression pattern like that of its homolog* Bra020735*, indicating that these genes might be conserved key regulator in cold stress responses. Moreover,* Bol033132* and* Bol042729* encode bZIP proteins that include the LCR containing amino acid N or N-rich region (Figure 5, Tables S4 and S5). These results indicated that the N-containing region of BolbZIP proteins might be involved in cold stress responses. Although the functions of the N-containing region are largely unknown, the regions might be biologically active [24, 25]. This genome-wide identification and expression profiling of bZIP proteins from* B. oleracea* provides new opportunities for functional analyses, which may be used in further studies for improving stress tolerance in plants.

## Supplementary Material

The supplementary materials contain 5 files, they are some important data supported to the methods and results of the presented study. These data make paper easier to read and understand.

## Figures and Tables

**Table 1:** 119 BolbZIP proteins were divided into 63 categories based on amino acid sequence similarity.

Index	*B. oleracea*			Identity 1 (%)	Identity 2 (%)	*B. rapa* homologs			*A. thaliana* homologs		
	Bol number	Length (aa)	Group			Bra number	Length (aa)	Group	At number	Length (aa)	Group
1	Bol000879	311	3A	95	75	Bra004550	379	3A	At2g46270	382	3A
	Bol017742	328	3A	80	70						
	Bol029580	300	11	76	79						

2	Bol004832	300	11	65, 98, 62, 62	75, 64	Bra000256	362	11	At2g42380	321	4A
	Bol001886	306	11	82, 75, 61, 62	71, 65	Bra004689	306	4B	At3g58120	329	4A
						Bra007380	318	4A			
						Bra003320	304	11			

3	Bol005115	343	1A	62, 83,92	83	Bra000195	334	1A	At2g40620	367	1A
	Bol006882	356	1A	59, 98, 86	79	Bra004582	356	1A			
	Bol020604	336	1A	88, 66, 67	66	Bra016980	342	1A			

4	Bol005139	617	10	79	53	Bra016959	624	10	At2g40950	721	10
	Bol006897	639	10	65	61						

5	Bol004200	281	12	59, 83, 84, 83, 60	64, 88	Bra004597	281	12	At2g41070	262	12
	Bol005146	272	12	74, 59, 60, 60, 93	69, 60	Bra007274	282	12	At3g56850	297	6B
	Bol006902	239	12	93, 59, 60, 61, 70	64, 58	Bra007276	282	12			
	Bol044306	289	6B	57, 80, 79, 90, 59	61, 77	Bra014668	229	12			
	Bol044413	278	12	58, 96, 95, 83, 60	65, 84	Bra016953	267	12			

6	Bol006077	392	11	94	71	Bra036251	394	2B	At4G02640	417	2B

7	Bol006734	270	5B	94		Bra030310	151	11			

8	Bol006735	425	5A	86		Bra030312	430	3A			

9	Bol006736	466	3A	93, 55	58	Bra030314	460	3A	At2g21230	525	5A
	Bol045878	372	3A	48, 90	50	Bra031172	376	3A			

10	Bol006975	149	11	95	74	Bra027855	149	12	At1g59530	148	12

11	Bol007295	334	11	94, 60, 90, 79, 94, 99	62, 92, 90, 81	Bra001443	331	11	At1g68640	452	11
	Bol010308	331	11	94, 62, 91, 81, 98, 94	65, 94, 91, 81	Bra004329	441	11	At3g12250	355	11
	Bol024000	442	11	61, 97, 61, 58, 62, 61	87, 55, 62, 59	Bra009241	310	11	At5g06950	330	11
	Bol024526	326	11	80, 58, 81, 99, 81, 80	60, 80, 81, 87	Bra028713	326	11	At5g06960	330	1A
	Bol035452	331	11	99, 61, 92, 80, 95, 94	64, 93, 90, 79	Bra034767	331	11			
	Bol043902	246	11	87, 57, 89, 76, 87, 86	59, 83, 89, 77	Bra038705	334	11			

12	Bol008040	380	11	67, 80, 68, 86, 57, 73	73, 77	Bra009063	364	12	At5g10030	364	12
	Bol009211	367	12	78, 98, 80, 91, 59, 82	81, 89	Bra024366	367	12	At5g65210	368	12
	Bol019052	390	12	75, 89, 77, 87, 74, 95	78, 86	Bra028604	362	12			
	Bol024636	362	12	88, 80, 99, 79, 50, 71	89, 78	Bra031871	370	12			
	Bol043707	364	12	97, 77, 87, 76, 46, 68	85, 75	Bra037374	314	11			
						Bra037809	392	12			

13	Bol008071	201	11	94, 54		Bra031845	136	12			
						Bra024424	249	11			

14	Bol008240	233	11	58, 90, 75, 60	62	Bra015471	392	1A	At1g06070	423	1A
	Bol023333	391	1A	73, 62, 56, 82	74	Bra018250	374	1A			
	Bol041035	342	1A	95, 62, 50, 73	77	Bra021735	339	11			
						Bra030637	381	1A			

15	Bol008830	102	12	77		Bra005971	160	11			

16	Bol009156	188	11	93	76	Bra033464	203	11	At3g51960	228	12

17	Bol009713	383	4A	85, 97	88	Bra016389	368	4A	At1g22070	384	11
						Bra031364	378	4A			

18	Bol010390	198	11	94, 83, 83	87	Bra019715	193	11	At1g13600	196	11
	Bol031441	195	11	78, 98, 98	84	Bra026895	195	11			
						Bra026896	195	11			

19	Bol010836	134	12	98, 81	78	Bra003500	134	12	At3g62420	146	12
	Bol044598	141	12	81, 98	88	Bra007679	141	12			
	Bol033132	171	11	97, 88	82, 56	Bra020735	171	11	At3g30530	173	11
	Bol043053	172	12	85, 98	88, 59	Bra025418	172	11	At5g38800	165	12

20	Bol011470	363	3A	95	69	Bra037382	367	3A	At4g01120	360	3A

21	Bol011683	96	12								
	Bol037733	106	12								

22	Bol011719	432	6A	92, 83	78	Bra005287	438	6A	At2g36270	442	6A
						Bra017251	396	6A			

23	Bol012142	160	6A	61, 95	61	Bra003755	179	6A	At1g75390	173	11
	Bol039324	160	6A			Bra008192	165	6A			
	Bol039895	178	6A	94, 62	76						

24	Bol012472	170	5B	97, 85, 84	79	Bra024478	155	5B	At2g18160	171	5B
	Bol041488	169	5B	86, 85, 96	83	Bra037235	168	5A			
						Bra039631	168	5A			

25	Bol012703	236	12	95, 72	73	Bra037290	239	12	At2g16770	249	12
	Bol042686	244	12	80, 89	71	Bra013048	239	12			

26	Bol013712	265	11	87, 63	61	Bra011580	231	12	At4g35040	261	4B
	Bol034645	255	11	64, 98	76	Bra034668	255	11			

27	Bol012855	294	6A	88	54	Bra033719	266	11	At5g44080	315	5B

28	Bol013623	416	1A	89	75	Bra011485	439	1A	At4g34000	454	1A
	Bol033853	410	11	71	55						

29	Bol013680	154	11	98, 89, 86	81	Bra011545	179	5B	At4g34590	159	5B
	Bol024237	148	5B	89, 97, 78	82	Bra017664	153	5B			
	Bol034676	142	11	84, 82, 98	78	Bra034639	142	11			

30	Bol014051	171	3A	66, 54, 66	68, 64	Bra005335	422	3A	At1g32150	389	3A
	Bol022259	422	3A	51, 50, 85	70, 62	Bra023012	403	3A	At2g35530	409	3A
	Bol027451	392	3A	96, 66, 64	63, 83	Bra023243	352	3A			
	Bol039799	400	3A	62, 85, 55	58, 73						

31	Bol015239	391	12	87		Bra033649	414	1A			

32	Bol016052	394	12	72		Bra010722	445	4B			

33	Bol016288	374	11	96, 74	72	Bra027885	373	1A	At1g58110	374	11
						Bra035464	176	1B			

34	Bol016432	289	11	79, 98	84	Bra009793	291	11	At5g24800	277	5B
	Bol022397	287	11	93, 77	81	Bra020471	289	11			

35	Bol016607	142	12	94	78	Bra010035	142	12	At5g49450	145	11
	Bol032354	139	12	80	76						

36	Bol003614	353	1A	80, 50, 70	54, 72	Bra001742	355	1B	At1g49720	403	1A
	Bol016788	307	1B	52, 87, 52	61, 48	Bra018800	368	1B	At3g19290	432	1A
	Bol018082	133	12	83, 64, 96	59, 91	Bra037533	388	1A			
	Bol031002	391	1A	74, 57, 88	53, 74						

37	Bol017068	187	12	93, 83, 73	60	Bra013005	182	12	At5g60830	206	12
	Bol036259	210	12	73, 85, 95	60	Bra029353	104	12			
						Bra035957	184	12			

38	Bol018521	442	1A	75	57	Bra033582	446	11	At4g38900	553	1A

39	Bol018596	243	1B	69, 95	70	Bra011780	246	1B	At4g37730	305	11
	Bol028894	246	1B	94, 66	72	Bra017850	240	1B			

40	Bol018688	281	11	73, 51, 92	67	Bra010504	222	11	At4g35900	285	5A
	Bol029042	270	5B	70, 66, 64	62	Bra011648	262	5A			
	Bol029939	265	11	90, 59, 66	66	Bra017735	259	5B			

41	Bol020032	89	11	78, 76, 100	82	Bra017359	174	11	At2g04038	166	11
	Bol032575	176	11	91, 81, 78	69	Bra025144	170	5B			
	Bol042729	170	5B	80, 97, 78	77	Bra026523	89	11			

42	Bol020390	389	11	88		Bra000102	366	11			

43	Bol021255	194	4B	77, 73, 97	79	Bra006324	181	4A	At5g15830	186	4A
	Bol034371	178	4A	93, 71, 82	75	Bra008670	183	4B			
	Bol030487	187	4B	70, 93, 75	73	Bra023540	188	4B			

44	Bol021964	190	12	64	64	Bra036025	190	12	At3g49760	156	12
	Bol037334	186	12	93	64						

45	Bol022925	148	5B	97, 84, 88	92	Bra001671	150	5B	At3g17609	149	5B
	Bol030865	145	5B	86, 97, 84	88	Bra021258	146	5B			
	Bol038660	150	11	81, 78, 96	83	Bra022225	116	12			

46	Bol023161	624	10	91, 87	59	Bra023224	593	10	At3g10800	675	10
						Bra034147	629	10			

47	Bol023356	318	5A	96, 84	80	Bra030663	320	5A	At1g06850	337	5A
						Bra031541	324	5A			

48	Bol024704	162	5B	85, 94	84	Bra008976	164	5A	At5g11260	168	5B
	Bol043589	164	5B	90, 88	87	Bra023317	166	5A			

49	Bol026864	459	11	97	74	Bra025743	462	11	At1g19490	471	11

50	Bol027526	791	12	97	83	Bra015646	339	12	At1g77920	368	1B

51	Bol027732	371	6A	67		Bra015847	358	6A			

52	Bol028631	120	11	97	73	Bra038341	120	12	At1g68880	138	12

53	Bol028975	313	3A	87	82	Bra010572	313	3A	At4g36730	315	3A
				96		Bra011701	313	3A			

54	Bol033486	303	11	65	55	Bra034925	233	2B	At1g42990	295	10

55	Bol033489	250	11	84, 96	70	Bra032191	330	3A	At1g43700	341	11
	Bol043246	330	3A	99, 82	69	Bra034916	263	11			

56	Bol033493	310	1B	97	71	Bra034913	222	1B	At1g35490	300	1A

57	Bol037803	266	11								

58	Bol040859	266	5A	93	64	Bra015281	268	5A	At1g03970	270	5A

59	Bol041278	333	11	93	80	Bra019436	336	11	At3g44460		12

60	Bol043859	149	12	97		Bra009288	147	12			

61	Bol044292	464	10	67		Bra014680	438	10			

62	Bol045190	385	4A	87	51	Bra040260	364	11	At1g45249	427	1A

63	Bol045877	386	3A	85		Bra031173	387	3A			

Length: amino acid length of bZIP proteins. Identity 1: homology between *B. oleracea* and *B. rapa*. Identity 2: homology between *B. oleracea* and *A. thaliana*.

**Table 2:** Number of bZIP transcription factors in each group based on UARR and LCRs.

Group	Classification domain	bZIP number in *B. oleracea*	bZIP number in *B. rapa* (Hwang et al.^*∗*^)	bZIP number in *A. thaliana* (Hwang et al.^*∗*^)
Group 1	Q-rich domain	13	16	10
Group 2	D-rich domain	0	4	3
Group 3	P-rich domain	12	12	6
Group 4	N-rich domain	5	9	4
Group 5	S-rich domain	13	18	14
Group 6	G-rich domain	7	6	2
Group 10	Transmembrane domain	4	4	4
Group 11	Several LCRs	38	41	17
Group 12	No LCR or UARR	27	26	13
Total	—	119	136	73

^*∗*^See reference [16].

**Table 3:** Cold-treatment induced change in expression based on RNA sequencing data. The differentially expressed genes determined based on fold change (FC) ≥2 are displayed with bold font and ≤0.5 with italic font.

Locus_ID	FC1		FC2		Contigs length (bp)	BRAD Bol number	CDS length (bp)	*A. thaliana* homologs	Published name
	(BN106)	*P* value	(BN107)	*P* value					
Locus_01882	**2.18 ± 0.11**	0.0031	1.48 ± 0.02	0.0053	1948	Bol009713	1152	AT1G22070	
Locus_01909	*0.16 ± 0.01*	0.0052	0.65 ± 0.05	0.0105	1555	Bol001886	921	AT2G42380	
Locus_04358	**3.84 ± 0.02**	0.0002	NC	NC	1583	Bol044598	426	AT3G62420	
Locus_05013	**4.58 ± 0.21**	0.0158	1.89 ± 0.51	0.0044	1207	Bol012472	513	AT2G18160	GBF5
Locus_06292	**2.35 ± 0.05**	0.0044	1.20 ± 0.03	0.0474	1081	Bol013712	798	AT4G35040	
Locus_08860	**13.09 ± 0.32**	0.0006	**2.08 ± 0.11**	0.0002	1541	Bol027526	2376	AT1G77920	TGA7
Locus_10723	**2.99 ± 0.19**	0.0012	**2.28 ± 0.35**	0.0077	1579	Bol026864	1380	AT1G19490	
Locus_10986	*0.06 ± 0.00*	0.0062	0.76 ± 0.04	0.0017	1117	Bol016607	429	AT5G49450	
Locus_11058	0.57 ± 0.03	0.0177	0.60 ± 0.04	0.0497	1354	Bol004832	903	AT2G42380	
Locus_11330	*0.27 ± 0.01*	0.0133	1.51 ± 0.33	0.0628	775	Bol042729	513	AT2G04038	
Locus_12559	*0.35 ± 0.01*	0.0090	0.87 ± 0.07	0.0994	1451	Bol028975	942	AT4G36730	GBF1
Locus_14643	0.83 ± 0.19	0.2500	*0.32 ± 0.10*	0.0346	816	Bol033132	516	AT3G30530	
Locus_14780	**4.80 ± 0.93**	0.0083	0.78 ± 0.03	0.0182	1882	Bol014051	516	AT1G32150	
Locus_15053	*0.15 ± 0.00*	0.0049	*0.47 ± 0.03*	0.0260	1757	Bol011470	1092	AT4G01120	GBF2
Locus_16059	**4.67 ± 2.83**	0.0358	1.29 ± 0.28	0.3559	873	Bol027732	1116		
Locus_18258	1.46 ± 0.81	0.3124	NC	NC	1013	Bol011719	1299	AT2G36270	ABI5
Locus_19284	*0.48 ± 0.02*	0.0023	1.00 ± 0.16	0.0535	1580	Bol006077	1179	AT4G02640	BZO2H1
Locus_19975	**5.14 ± 0.02**	0.0015	**3.11 ± 0.24**	0.0000	1113	Bol028894	741	AT4G37730	
Locus_20038	**2.25 ± 0.04**	0.0002	0.74 ± 0.09	0.0017	2250	Bol033853	1233	AT4G34000	ABF3
Locus_21455	**2.15 ± 0.03**	0.0012	1.32 ± 0.05	0.0174	1248	Bol041488	510	AT2G18160	GBF5
Locus_22202	**2.90 ± 0.22**	0.0078	0.67 ± 0.03	0.0061	1566	Bol000879	936	AT2G46270	GBF3
Locus_22929	*0.27 ± 0.05*	0.0569	0.58 ± 0.11	0.0236	890	Bol037803	801		
Locus_25534	**7.11 ± 1.40**	0.0024	1.87 ± 0.19	0.0400	1165	Bol039895	537	AT1G75390	
Locus_27120	*0.13 ± 0.16*	0.0645	NC	NC	545	Bol008071	606		
Locus_28516	NC	NC	NC	NC	284	Bol033493	933	AT1G35490	
Locus_31552	*0.29 ± 0.05*	0.0628	NC	NC	329	Bol006902	720	AT2G41070	DPBF4
Locus_31870	**6.75 ± 3.18**	0.0743	0.51 ± 0.17	0.1589	386	Bol037733	321		
Locus_35274	0.57 ± 0.02	0.0027	*0.19 ± 0.22*	0.0014	1107	Bol016432	870	AT5G24800	BZO2H2
Locus_35336	*0.12 ± 0.00*	0.0113	*0.17 ± 0.04*	0.0006	969	Bol021255	585	AT5G15830	
Locus_35982	**4.94 ± 0.07**	0.0010	**3.61 ± 0.30**	0.0016	1216	Bol034676	429	AT4G34590	ATB2/GBF6
Locus_36644	*0.40 ± 0.06*	0.0362	0.70 ± 0.07	0.1179	1588	Bol008040	1143	AT5G65210	TGA1
Locus_38207	0.56 ± 0.06	0.0396	*0.18 ± 0.21*	0.0272	673	Bol005115	1032	AT2G40620	
Locus_38300	1.23 ± 0.64	0.5000	*0.00 *	NC	318	Bol018596	732	AT4G37730	
Locus_38533	**4.51 ± 0.34**	0.0023	0.85 ± 0.09	0.0454	1978	Bol043707	1095	AT5G10030	TGA4
Locus_38636	**9.75 ± 0.87**	0.0272	0.56 ± 0.05	0.0346	487	Bol030865	438	AT3G17609	HYH
Locus_39177	1.20 ± 0.18	0.1749	*0.38 ± 0.01*	0.0054	839	Bol043589	495	AT5G11260	HY5
Locus_39837	0.78 ± 0.05	0.0267	**2.20 ± 1.21**	0.0097	1648	Bol041035	1038	AT1G06070	
Locus_39980	NC	NC	NC	NC	478	Bol038660	453	AT3G17609	HYH
Locus_41080	*0.07 ± 0.04*	0.0033	**2.32 ± 0.80**	0.0840	677	Bol010390	597	AT1G13600	
Locus_44632	NC	NC	NC	NC	256	Bol029939	798	AT4G35900	FD-1
Locus_44950	**2.86 ± 0.00**	0.0052	1.32 ± 0.05	0.0301	1447	Bol024526	981	AT5G06960	TGA5/OBF5
Locus_45018	NC	NC	0.70 ± 0.02	0.0223	667	Bol022925	447	AT3G17609	HYH
Locus_46951	*0.15 ± 0.17*	0.0257	NC	NC	462	Bol020032	270	AT2G04038	
Locus_47897	NC	NC	NC	NC	458	Bol037334	561	AT3G49760	
Locus_49075	*0.39 ± 0.10*	0.0149	**2.76 ± 0.40**	0.0145	739	Bol034371	537	AT5G15830	
Locus_55049	NC	NC	0.57 ± 0.29	0.1464	311	Bol018688	846	AT4G35900	FD-1
Locus_56035	*0.04 ± 0.00*	0.0010	*0.15 ± 0.06*	0.0055	662	Bol032354	420	AT5G49450	

NC, not calculated. FC1, signal intensity of 0°C treated plant over control plant (22°C) in *BN106*. FC2, signal intensity of 0°C treated plant over control plant in *BN107*.
